# Anti‐Coronaviral Nanocluster Restrain Infections of SARS‐CoV‐2 and Associated Mutants through Virucidal Inhibition and 3CL Protease Inactivation

**DOI:** 10.1002/advs.202207098

**Published:** 2023-02-26

**Authors:** Hao Tang, Hongbo Qin, Shiting He, Qizhen Li, Huan Xu, Mengsi Sun, Jiaan Li, Shanshan Lu, Shengdong Luo, Panyong Mao, Pengjun Han, Lihua Song, Yigang Tong, Huahao Fan, Xingyu Jiang

**Affiliations:** ^1^ Shenzhen Key Laboratory of Smart Healthcare Engineering Guangdong Provincial Key Laboratory of Advanced Biomaterials Department of Biomedical Engineering Southern University of Science and Technology Shenzhen Guangdong 518055 P. R. China; ^2^ Beijing Advanced Innovation Center for Soft Matter Science and Engineering College of Life Science and Technology Beijing University of Chemical Technology Beijing 100029 P. R. China; ^3^ Institute of Chemical Biology Shenzhen Bay Laboratory Shenzhen Guangdong 518055 P. R. China; ^4^ The Fifth Medical Center Chinese People's Liberation Army General Hospital Beijing 100039 P. R. China

**Keywords:** 3CL protease, antiviral, coronavirus, nanoclusters, virucidal inhibition

## Abstract

Antivirals that can combat coronaviruses, including SARS‐CoV‐2 and associated mutants, are urgently needed but lacking. Simultaneously targeting the viral physical structure and replication cycle can endow antivirals with sustainable and broad‐spectrum anti‐coronavirus efficacy, which is difficult to achieve using a single small‐molecule antiviral. Thus, a library of nanomaterials on GX_P2V, a SARS‐CoV‐2‐like coronavirus of pangolin origin, is screened and a surface‐functionalized gold nanocluster (TMA‐GNC) is identified as the top hit. TMA‐GNC inhibits transcription‐ and replication‐competent SARS‐CoV‐2 virus‐like particles and all tested pseudoviruses of SARS‐CoV‐2 variants. TMA‐GNC prevents viral dissemination through destroying membrane integrity physically to enable a virucidal effect, interfering with viral replication by inactivating 3CL protease and priming the innate immune system against coronavirus infection. TMA‐GNC exhibits biocompatibility and significantly reduces viral titers, inflammation, and pathological injury in lungs and tracheas of GX_P2V‐infected hamsters. TMA‐GNC may have a role in controlling the COVID‐19 pandemic and inhibiting future emerging coronaviruses or variants.

## Graphical abstract

A mixed‐ligand protected gold nanocluster as the potent nano‐antiviral for combating coronaviruses is reported, including SARS‐Cov‐2 and its variants through physical and biological mechanisms. The anti‐coronaviral gold nanocluster not only holds promise in countering the pandemic SARS‐CoV‐2 and future emerging coronaviruses but will also benefit the discovery of novel broad‐spectrum macromolecular antivirals.

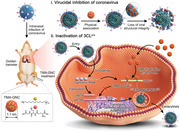

## 1 Introduction

Severe acute respiratory syndrome coronavirus 2 (SARS‐CoV‐2) was the third global coronavirus outbreak of coronavirus in humans after SARS‐CoV (2003) and the Middle East respiratory syndrome (MERS, 2012).^[^ ^1^, ^2^ ^]^ SARS‐CoV‐2 was an enveloped, single‐stranded, positive‐sense RNA virus belonging to the family *Coronaviridae*.^[^ ^3^, ^4^ ^]^ SARS‐CoV‐2 caused more than 561 million confirmed cases of novel coronavirus 2019 (COVID‐19) in over 200 countries and territories, and more than 6.5 million deaths as of September 2022 (worldometers.info/coronavirus/). Vaccination was the most effective strategy to protect people from viral infections; however, problems such as incomplete protection and unidentified long‐term efficacy remained.^[^ ^5^, ^6^ ^]^ Specific antivirals combined with effective vaccination were believed to be promising solutions to end the COVID‐19 pandemic.^[^ ^7^, ^8^, ^9^, ^10^, ^11^, ^12^, ^13^ ^]^ Presently, two oral antivirals, Paxlovid from Pfizer and Molnupiravir from Merck, were approved by the U.S. Food and Drug Administration to treat COVID‐19.^[^ ^14^, ^15^ ^]^ Nowadays, the accessibility of broad‐spectrum antivirals against coronaviruses, including SARS‐CoV‐2, SARS‐CoV‐2 variants of concern (VOC) and emerging coronaviruses in the future, was still limited.^[^ ^12^, ^16^ ^]^ SARS‐CoV‐2 evolved into susceptible VOCs, such as Delta (B.1.617.2) and Omicron (B.1.1.529) variants, raising challenges regarding increased transmissibility and virulence of the disease^[^ ^17^ ^]^ and decreased effectiveness of available vaccines or therapeutics.^[^ ^18^, ^19^ ^]^ Therefore, it was urgently needed to explore new therapeutic agents with broad‐spectrum antiviral capabilities against pathogenic coronaviruses and their variants.

The native structure and replication cycle of coronaviruses should be accounted for when considering therapeutic options. Structurally, ribonucleoproteins of coronavirus were highly ordered and assembled inside the surrounding lipid bilayer of coronavirus.^[^ ^20^ ^]^ The viral lipid bilayer was fundamentally derived from host cell components and functioned as a structural building block for molecular signaling.^[^ ^20^, ^21^, ^22^ ^]^ During viral infection, the coronavirus underwent multiple steps, including attachment/entry, replication, assembly and release.^[^ ^16^ ^]^ After entry into host cells, coronaviruses uncoated, translated proteins, and replicated genomic RNA for virion assembly and exocytosis. In particular, coronavirus transcription and replication required the participation of the main viral protease (also known as 3C‐like protease, 3CL^pro^), which mediated the cleavage of large replicase polyprotein 1a (pp1a) and pp1ab into non‐structural proteins.^[^ ^23^, ^24^, ^25^ ^]^ 3CL^pro^ had no human homologue and was highly conserved among known coronavirus species; thus, 3CL^pro^ was a mechanistically safe and broad‐spectrum target for antiviral treatments.^[^ ^26^, ^27^ ^]^ Generally, antiviral candidates were designed to target a certain host‐based or viral‐based therapeutic option throughout the life cycle of coronavirus;^[^ ^16^ ^]^ however, the discovery of antiviral agents that inactivated the virus and inhibited intracellular viral replication simultaneously was rarely reported. This approach could enable the use of candidates with multiple modes of action and sustainable, enhanced, and broad‐spectrum antiviral efficacies. Inspired by the fact that nanoparticles (NPs) were typically used as disinfectants to prevent the dissemination of pathogens, including viruses and bacteria, through diverse mechanisms of action,^[^ ^28^, ^29^ ^]^ we proposed the possibility of investigating an antiviral nanodrug that could not only physically abolish the vitality of coronaviruses but also interfere with the intracellular process of viral replication.

Nanoparticles had been adopted to prevent and treat infectious diseases by taking advantage of their programmable anti‐pathogenic properties.^[^ ^30^, ^31^, ^32^ ^]^ Indeed, an increasing number of studies had reported the employment of nanomaterials as antivirals, including metallic nanoparticles, carbon dots, polymeric nanocapsules and bio‐mimicking nanoparticles.^[^ ^33^, ^34^, ^35^, ^36^, ^37^, ^38^ ^]^ Among them, some gold nanoparticles could block the interaction between viruses and host cells by multivalent effects of the surface capping ligands.^[^ ^39^, ^40^, ^41^ ^]^ However, no studies reported the employment of a single gold nanomaterial to be an anti‐coronaviral nano‐cocktail treatment with multiple modes of action. Besides, the in vivo anti‐coronaviral efficacy of gold nanomaterials was in lacking so far. Regarding COVID‐19, we proposed that nano‐antivirals would execute their activity through multiple mechanisms of action, providing broad‐spectrum antiviral activity against SARS‐CoV‐2 and its variants. However, the use of nanomaterials had raised concerns about their potential toxicity. Thus, exploring effective and safe nano‐antivirals that combat the SARS‐CoV‐2 pandemic and future emerging coronaviruses is extremely important and remains a challenge.

**Scheme 1:**
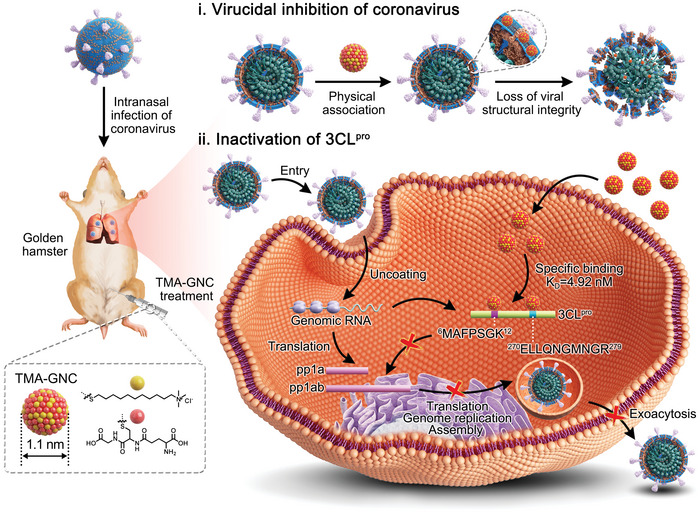
Summary of antiviral properties of TMA‐GNC, which inhibited coronavirus infection by disrupting the structural integrity and inactivating the virus‐associated 3CL^pro^.

Here, we demonstrated an anti‐coronaviral nanocluster, which killed coronaviruses through physical association and inhibited viral propagation by inactivating viral 3CL^pro^ (**Scheme** **1**). Physical virucidal interaction was achieved by associating the lipid components of the viral membrane with the aid of quaternary ammonia heads on the surface of the nanocluster. At the same time, the second zwitterionic ligand prevented such efficacy in cells (e.g., erythrocytes). In addition, the nanocluster functioned as a high‐affinity binder and inhibitor of 3CL^pro^ and efficiently blocked subsequent biological processes, including translation, genome replication, budding and assembly, and exocytosis. The nanocluster was shown to effectively counter coronavirus infection in vivo with minimal biological toxicity. Overall, these results illustrated the versatility of nanomedicine in developing effective antivirals and combatting the coronavirus pandemic, which can be harnessed as a therapeutic option.

## 2 Results and Discussion

### 2.1 Identification of the Anti‐Coronaviral Nanocluster

**Figure 1:**
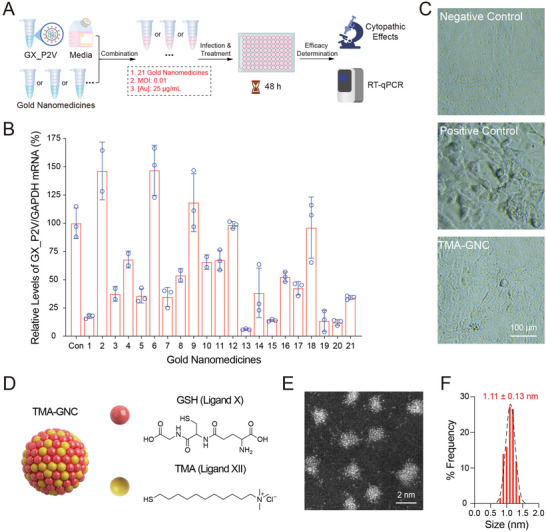
Identification of TMA‐GNC as a potent anti‐coronaviral nanodrug. A) Schematic diagram of the single dosage screening assay of antiviral nanomedicines using SARS‐CoV‐2‐like GX_P2V as the model virus. B) Viral RNA level analysis of GX_P2V infected Vero E6 cells after treatment with antiviral candidates for 48 h. Each column represented a nanodrug; 1–19 were gold nanomaterials that modified with different surface ligands as illustrated in Figure S1, Supporting Information; 20 (AuRh) and 21 (AuRu) were bimetallic nanoparticles.^[^ ^48^ ^]^ C) TMA‐GNC treatment significantly inhibited the cytopathic effects of infected cells, as observed through an inverted microscope. D) Schematic illustration of TMA‐GNC functionalized with mixed ligands of glutathione (GSH, ligand 10, red) and *N*,*N*,*N*‐trimethyl(11‐mercaptoundecyl) ammonium chloride (TMA, ligand 12, blue). E) High‐resolution scanning transmission electron microscopy images of TMA‐GNC. F) Statistical analysis of the size distribution of the TMA‐GNC (*n* = 300).

In this study, we used the SARS‐CoV‐2‐like coronavirus GX_P2V, an ideal alternative drug screening model that shared 86% genome nucleotide sequence and 92.2% amino acid identity in spike protein with SARS‐CoV‐2,^[^ ^4^, ^9^ ^]^ as the virus model to screen anti‐coronaviral nanomedicines. Nanomedicines employed in the screening experiments included 19 gold nanoparticles/nanoclusters with a diverse capping of surface ligands (Figure S1, Supporting Information) and two bimetallic NPs (AuRh and AuRu). These nano drugs had been reported to be effective in inhibiting the growth of bacteria in our previous works.^[^ ^42^, ^43^, ^44^, ^45^, ^46^, ^47^, ^48^, ^49^ ^]^ Vero E6 cells were infected with GX_P2V at an MOI of 0.01, and drug candidates were added to reach a final concentration of 25 µg mL^−1^ (gold) for single‐dose screening. Cytopathic effects (CPE) were observed, and RT‐qPCR analysis was performed to determine the antiviral effects of the drugs (**Figure** **1**A). Among tested nanomaterials, the mixed glutathione (GSH, ligand X) and *N*,*N*,*N*‐trimethyl (11‐mercaptoundecyl) ammonium chloride (TMA, ligand XII)‐protected gold nanoclusters, abbreviated as TMA‐GNC, exhibited the most potent anti‐GX_P2V efficacy with a 94% decrease in GX_P2V/GAPDH mRNA level (Figure 1B). Consistently, we observed the absence of CPE caused by virus infection in the microscopic images of TMA‐GNC‐treated infected cells (Figure 1C).

TMA‐GNC was prepared by reducing chloroauric acid with GSH under mild hydrothermal conditions (70 °C, 24 h). We optimized the feeding ratio of TMA/GSH/HauCl_4_ to 2:2:1 to obtain a stable formulation for dialysis purification and long‐term storage. TMA‐GNC appeared to be a colorless and transparent solution (Figure S2A, Supporting Information) and remained unchanged in appearance during one year of storage at 4 °C. TMA‐GNC showed absorbance within 400 nm in the UV–Vis spectrum and exhibited luminescence with maximum excitation and emission peaks at 375 and 610 nm, respectively (Figure S2B, Supporting Information). We applied double spherical aberration‐corrected TEM (DSAC‐TEM) to analyze the size of TMA‐GNC, which had an average diameter of 1.11 nm (Figure 1E,F). Energy‐dispersive X‐ray spectrometry (EDS) analysis confirmed the distribution of mercaptan ligands on the surface of TMA‐GNC (Figure S2C, Supporting Information). Based on X‐ray photoelectron spectroscopy and nuclear magnetic resonance analysis, we determined that each GNC contained 42 Au atoms, 39 GSHs and 16 TMAs (Figure S2D, Supporting Information). In an aqueous solution, TMA‐GNC exhibited a hydrodynamic diameter of 4.79 ± 0.43 nm and surface potential of +14.7 ± 2.72 mV (Figure S2E,F, Supporting Information).

### 2.2 Anti‐Coronaviral Efficacy of TMA‐GNC In Vitro

**Figure 2:**
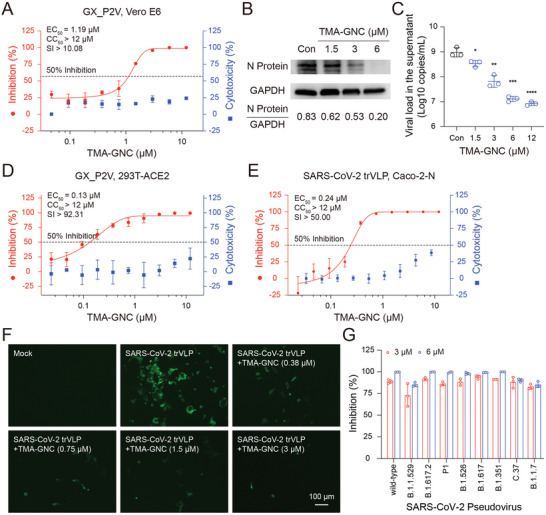
Evaluation of anti‐coronavirus efficacy of TMA‐GNC in different in vitro models. A) Inhibition analysis of GX_P2V infection on Vero E6 cells by different doses of TMA‐GNC. B) Western blot analysis of the amount of nucleocapsid after treatment with TMA‐GNC. C) RT‐qPCR analysis of inhibition of extracellular GX_P2V production after treatment with TMA‐GNC. D) Inhibition analysis of GX_P2V infection on angiotensin‐converting enzyme 2‐overexpressing 293T cells (293T‐ACE2) by different doses of TMA‐GNC. E) Inhibition analysis of SARS‐CoV‐2 trVLP by TMA‐GNC on Caco‐2‐N cells. For (A), (D), and (E), the left and right Y‐axis represented the percentage of inhibition of virus yield (Inhibition, %) and cytotoxicity against used host cell lines (Cytotoxicity, %), respectively. F) Observation of GFP signals of SARS‐CoV‐2 GFP/ΔN trVLP by fluorescence microscope after treatment with TMA‐GNC. G) Inhibition of SARS‐CoV‐2 variants of concerns and variants of interests by TMA‐GNC (3 and 6 µm).

In the concentration‐dependent assay, TMA‐GNC effectively inhibited the GX_P2V infection in Vero E6 cells with a 50% effective concentration (EC_50_) of 1.19 µm (**Figure** **2**A). The EC_50_ value of TMA‐GNC was lower than that of nirmatrelvir (PF‐07321332, 3.36 µm) and Aluvia (lopinavir and ritonavir, 2.71 µm), and comparable to that of remdesivir (GS‐5734, 1.01 µm), demonstrating the potent antiviral efficacy of TMA‐GNC (Figure S3, Supporting Information). The amount of viral nucleoprotein (N protein) significantly decreased with increasing doses of TMA‐GNC (Figure 2B). The virus yield was determined by analyzing RNA copies and 50% tissue culture infective dose (TCID_50_) values of the supernatants of infected cells. TMA‐GNC effectively inhibited the production of virions, with significant decreases in viral titres (Figure 2C) and median tissue culture infectious dose (TCID_50_, Figure S4, Supporting Information) by 2‐ and 3‐log values, respectively. The 50% cytotoxic concentration (CC_50_) of TMA‐GNC against Vero E6 cell lines was over 12 µm, which enabled the selectivity index (SI) to exceed 10.08. We assessed the anti‐GX_P2V effects of TMA‐GNC using the angiotensin converting enzyme‐2 (ACE2)‐overexpressing 293T cell line (293T‐ACE2) as host cells. The EC_50_ and CC_50_ were determined to be 0.13 µm and higher than 12 µm, respectively, and the SI was over 92.31 (Figure 2D). We also tested TMA‐GNC against porcine epidemic diarrhea virus (PEDV) and swine acute diarrhea syndrome coronavirus (SADS‐CoV). The EC_50_ were determined to be 0.09 (PEDV) and 0.17 µm (SADS‐CoV), respectively (Figure S5, Supporting Information). We also found that TMA‐GNC showed negligible antiviral efficacy to the rotavirus which was a non‐enveloped virus (Figure S6, Supporting Information). A SARS‐CoV‐2 life cycle‐mimicking cell culture system with transcription‐ and replication‐competent SARS‐CoV‐2 virus‐like‐particles (SARS‐CoV‐2 trVLP) was employed to evaluate the anti‐SARS‐CoV‐2 ability of TMA‐GNC.^[^ ^50^ ^]^ The EC_50_ of TMA‐GNC was 0.24 µm against SARS‐CoV‐2 trVLP, demonstrating that TMA‐GNC was a potent antiviral against SARS‐CoV‐2 infection with an SI higher than 50 (Figure 2E). Consistently, in the SARS‐CoV‐2 trVLP system, in which the viral nucleocapsid gene was replaced by the reporter gene (green fluorescent protein, GFP), we observed a significant decrease in green fluorescent protein (GFP) signals of the trVLP upon treatment with TMA‐GNC (Figure 2F). We next evaluated the antiviral activity of TMA‐GNC toward SARS‐CoV‐2 related mutant strains using a pseudotyped virus‐based assay.^[^ ^51^ ^]^ As shown in Figure 2G, excellent activity against SARS‐CoV‐2 pseudoviruses, including VOCs (B.1.1.529, B.1.351, P.1, B.1.1.7, and B.1.617.2) and variants of interest (VOI, B.1.526, and C.37), were determined. An inhibition rate of almost 100% of TMA‐GNC was achieved against all tested pseudoviruses. Altogether, these results demonstrated the potent and broad‐spectrum efficacy of TMA‐GNC in inhibiting the infection, replication and production of coronaviruses, including SARS‐CoV‐2‐like GX_P2V, SARS‐CoV‐2 trVLP and SARS‐CoV‐2 variant pseudoviruses.

### 2.3 Anti‐Coronaviral Mechanisms of TMA‐GNC

**Figure 3:**
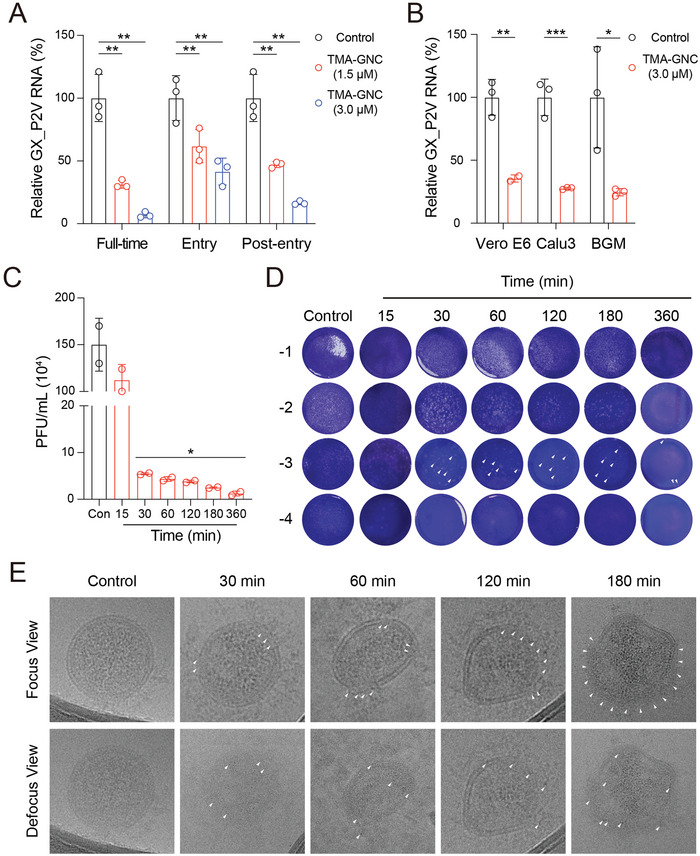
Physical association between TMA‐GNC and coronavirus enabled the GNC with virucidal activity. A) Inhibition of GX_P2V infection by TMA‐GNC during full‐time, entry, and post‐entry processes. B) Inhibition of GX_P2V infection in Vero E6, Calu3, and BGM cells after virus pre‐incubation with TMA‐GNC. (C) and (D) time‐dependent virucidal activity of TMA‐GNC against GX_P2V tested by plaque‐forming assay. The significant decreases in the number of plaques were observed after the 30 min treatments. The white arrows indicated the plaques. E) Unstained Cryo‐EM images of GX_P2V after direct association with TMA‐GNC for a different duration. The white arrows in the upper and lower panel indicated the integrity loss of viral membrane structures and representative TMA‐GNC that interacted with the coronaviruses, respectively.

Having established anti‐coronavirus activity, we studied the mechanisms of action of TMA‐GNC during the coronavirus life cycle. In time‐of‐addition experiments, TMA‐GNC blocked viral entry and inhibited viral post‐entry (**Figure** **3**A and Figure S7, Supporting Information). This indicated multiple anti‐coronavirus mechanisms of TMA‐GNC. First, we confirmed the influence of direct viral attachment on the antiviral efficacy of the TMA‐GNC. After challenging host cells with the pre‐incubated TMA‐GNC and GX_P2V, we observed clear inhibition of GX_P2V infection in different host cell lines, including Vero E6, Calu3 and BGM (Figure 3B). In the virucidal assay, the number of live viruses decreased significantly when we pre‐treated GX_P2V with TMA‐GNC before viral infection, confirming the virucidal activity of TMA‐GNC through physical viral association (Figure 3C,D). To investigate the mechanism of virucidal inhibition, we studied the physical interactions between the TMA‐GNC and viral nanoparticles. Through energy‐dispersive X‐ray spectrometry (EDS) analysis, we observed the accumulation and distribution of gold around the surface of GX_P2V after incubation with TMA‐GNC and viral nanoparticles (Figure S8, Supporting Information). Because GX_P2V was an enveloped virus surrounded by a lipid bilayer like the SARS‐Cov‐2,^[^ ^22^ ^]^ we speculated that the physical contact was attributed to the fact that quaternary ammonium groups from the TMA ligand enabled the insertion of TMA‐GNC into the bilayer structure of virions, causing integrity loss of the viral structure. To verify this aspect, we used cryo‐EM to study the impact on the physical structure of coronaviruses upon TMA‐GNC treatment. The control group without TMA‐GNC association remained intact and spherical, exhibiting a clear bilayer structure. In contrast, we observed that TMA‐GNC interacted with virus particles, and TMA‐GNC accumulated on the surfaces of virus particles as the incubation time increased. TMA‐GNC damaged the viral membrane bilayer and disrupted viral structural integrity, exhibiting the physical and virucidal properties (Figure 3E).

**Figure 4:**
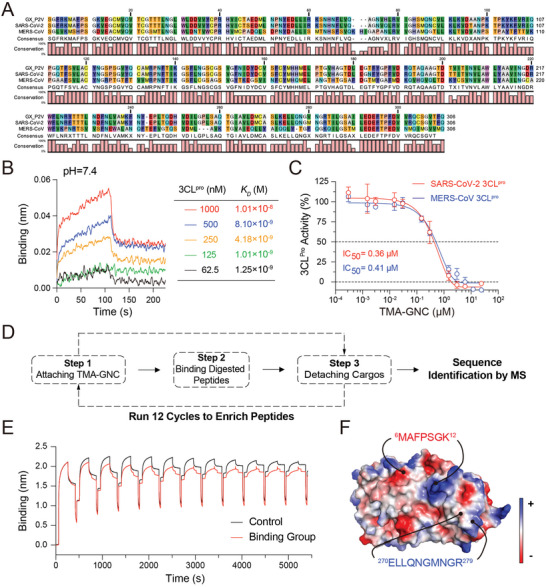
TMA‐GNC inhibited 3CL^pro^ enzymatic activity. A) The sequence alignment of 3CL^pro^ of GX_P2V, SARS‐CoV‐2, and MERS‐CoV. B) The binding kinetics of TMA‐GNC and SARS‐CoV‐2 3CL^pro^ in PBS. C) Dose‐response curves for the inhibition of SARS‐CoV‐2 3CL^pro^ and MERS‐CoV 3CL^pro^ by TMA‐GNC. D) Schematic diagram of applying Bio‐Layer Interferometry and mass spectrometry (MS) to enrich and identify targeting peptides of TMA‐GNC, respectively. E) Real‐time monitoring of the enrichment of target peptides onto biosensors in 12 cycles. F) The electrostatic potential map of SARS‐CoV‐2 3CL^pro^, with MS‐identified sequences marked at corresponding positions.

Next, we investigated the anti‐replication mechanism of TMA‐GNC in the post‐entry stage. Since TMA‐GNC showed broad anti‐coronavirus capabilities against GX_P2V and SARS‐CoV‐2, we proposed 3CL^pro^ as a possible intracellular target of TMA‐GNC. During the life cycle of coronaviruses, 3CL^pro^ was indispensable for viral replication and was a highly conserved protease among coronaviruses^[^ ^7^ ^]^ (**Figure** **4**A). The Bio‐Layer Interferometry (BLI) technology was applied to analyze the binding kinetics between TMA‐GNC and SARS‐CoV‐2 3CL^pro^. As shown in Figure 4B and Figure S9, Supporting Information, TMA‐GNC exhibited exceptional binding affinity to 3CL^pro^ with a dissociation constant (*K*_D_) ranging from 1.25 × 10^−9^ to 1.01 × 10^−8^ m, implying that TMA‐GNC was a high‐affinity binder of 3CL^pro^. We also tested the binding kinetics between TMA‐GNC and SARS‐CoV‐2 papain‐like protease (PL^pro^). Results showed that TMA‐GNC had no binding affinity to PL^pro^ (Figure S10, Supporting Information), implying that TMA‐GNC might behaved specifically against 3CL^pro^. We then tested the IC_50_ value (drug concentration that caused a 50% reduction in enzymatic activity) of TMA‐GNC to SARS‐CoV‐2 3CL^pro^ by a FRET reporter assay using ebselen as the positive control (Figure S11, Supporting Information). The IC_50_ value of TMA‐GNC was determined to be 0.36 µm (Figure 4C) and slightly higher than the EC_50_ value (0.24 µm, Figure 2E), suggesting that TMA‐GNC inhibition of coronavirus infection was achieved through a combined mechanism that included inactivating 3CL^pro^. The IC_50_ value of TMA‐GNC inhibition of MERS‐CoV 3CL^pro^ was 0.41 µm (Figure 4C), indicating the broad‐spectrum antiviral efficacy of TMA‐GNC against coronaviruses by inhibiting 3CL^pro^ activity.

To obtain a detailed view of the target sequences of TMA‐GNC against 3CL^pro^, we applied BLI technology to enrich the target peptides from a trypsin‐digested peptidyl library and used mass spectrometry (MS) to identify the enriched sequences (Figure 4D). In BLI experiments, we found that the binding response between TMA‐GNC and SARS‐CoV‐2 3CL^pro^ increased when acetic acid was used to lower the pH value of the binding buffer (Figure S12A, Supporting Information), and *K*_D_ values at the picometers level in a more acidic buffer (pH = 4.5) were identified (Figure S12B, Supporting Information). Thus, we ran 12 cycles of peptide enrichment in an acidic buffer (pH = 4.5) until the binding content of the biosensors became saturated (Figure 4E). According to the monitoring curves, the enrichment of target sequences decreased the electrostatic affinity of TMA‐GNC toward the biosensors, which were alginic acid‐modified and negatively charged. This implied that the binding peptides of TMA‐GNC should be positively charged in the binding buffer with isoelectric points above 4.5. Consistently, we identified two peptides that accessed SARS‐CoV‐2 3CL^pro^ with high confidence by MS, whereas no peptide was detected in the control group. The annotated sequences were MAFPSGK at positions 6–12 and ELLQNGMNGR at 270–279 in 3CL^pro^ (Figure 4F). Altogether, these results demonstrated that TMA‐GNC inhibited coronavirus infection through a combined mechanism, disrupting the viral membrane and inactivating 3CL^pro^. The targets of TMA‐GNC were highly conserved among coronavirus species; thus, the dual‐mechanism activity made TMA‐GNC a promising antiviral with broad‐spectrum efficacy against coronaviruses.

**Figure 5:**
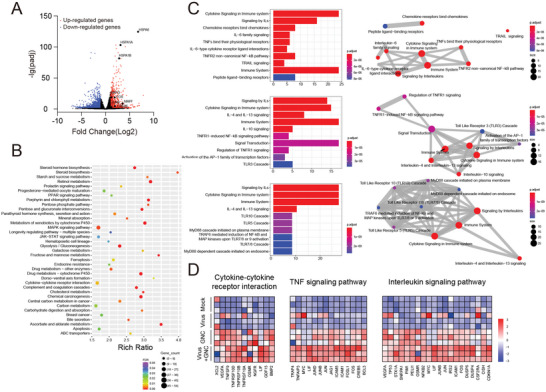
Transcriptional analysis after TMA‐GNC treatment of SARS‐CoV‐2 trVLP infection in Caco‐2‐N cells. A) Volcano map of total expression genes. Upregulated genes were in red, while downregulated genes were in blue. Labelled genes were from MAPK pathways (HSPA1A, HSPA1B, and HSPA6) and immediate‐early genes (FOS, MAFF, MAFG, JUNB, CREB5). B) The bubble plot of enriched pathways from all regulated genes in virus‐infected cells after TMA‐GNC treatment. C) Enriched pathways of all regulated genes from cytokine–cytokine receptor interaction (top), TNF signalling pathway (middle), and interleukin signalling pathway (bottom). The right row represents the network of these enriched items. D) Heatmap of exemplary upregulated genes from cytokine–cytokine receptor interaction, TNF signalling pathway, and interleukin signalling pathway.

Next, we performed RNA sequencing to profile transcriptional changes in SARS‐CoV‐2 trVLP‐infected Caco‐2‐N cells after TMA‐GNC treatment. Compared to the virus‐infected cells, there were a total of 3022 genes that changed significantly with |log_2_(fold change)| ≥ 1 and q(adjusted *p*‐value) < 0.05, including 1021 upregulated genes and 2001 downregulated genes (**Figure** **5**A). Kyoto Encyclopedia of Genes and Genomes (KEGG) pathway analysis showed that these genes were enriched in pathways including energy metabolism (glycolysis/gluconeogenesis, ascorbate and aldarate metabolism, fructose and mannose metabolism) and endocrine system (retinol metabolism, cholesterol metabolism) (Figure 5B). Evidence had shown that metabolic processes were associated with antiviral immune responses. Indeed, we observed the enrichment of upregulated genes in antiviral innate immunity‐related pathways, including cytokine–cytokine receptor interactions, TNF signalling pathway, interleukin signalling pathway, NF‐kB signalling pathway and mitogen‐activated protein kinase (MAPK). Further analysis revealed that these genes were enriched in the immune system, including cytokine signalling and signalling by interleukins (Figure 5C,D). In addition, we observed the upregulation of immediate‐early genes (e.g., FOS, MAFF, CREB5, and JUNB) in response to TMA‐GNC treatment (Figure 5A). These results implied that TMA‐GNC helped regulate the transcriptional landscape to modulate antiviral immune responses against coronavirus infection.

### 2.4 Biosafety Evaluation of TMA‐GNC

**Figure 6:**
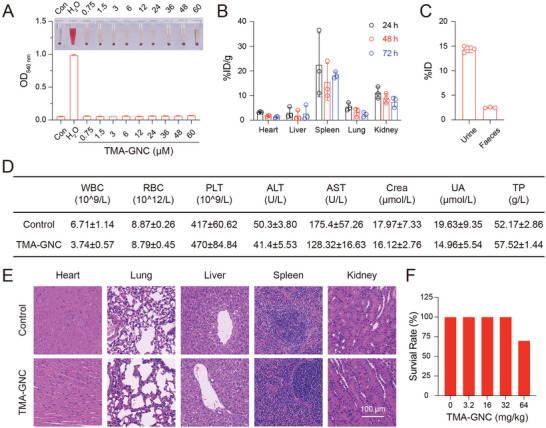
Evaluation of blood compatibility, biodistribution, and in vivo toxicity of TMA‐GNC. A) Hemolysis activity of TMA‐GNC. B) Biodistribution of TMA‐GNC in heart, liver, spleen, lung, and kidney. C) The amount of cleared TMA‐GNC in urine and feces 24 h after injection. D) Statistical analysis of hematological parameters (white blood cells, WBC; red blood cells, RBC; platelets, PLT) and serum chemistry indicators (alanine transferase, ALT; aspartate aminotransferase, AST; creatinine, Crea; uric acid, UA; total protein, TP) after i.p. treatment of TMA‐GNC (10 mg kg^−1^). E) Hematoxylin and eosin (H&E) staining of the heart, lung, liver, spleen, and kidney of mice after i.p. treatment with TMA‐GNC. F) The survival rate of mice after i.p. treatment of different doses of TMA‐GNC at 24 h (*n* = 10).

No hemolytic effects were observed even when erythrocytes were directly exposed to TMA‐GNC at the high concentration of 60 µm, demonstrating the high blood compatibility of TMA‐GNC (**Figure** **6**A). The cell cytotoxicity of TMA‐GNC was tested, and more than 90% of human umbilical vein endothelial cells (HUVEC) and human aortic fibroblasts (HAF) survived below 12 µm (Figure S13, Supporting Information). We used ICP‐MS to determine the biodistribution of TMA‐GNC in major organs, including the heart, liver, spleen, lung and kidney. After intraperitoneal (i.p.) administration of TMA‐GNC (10 mg kg^−1^) in BALB/c mice, the TMA‐GNC mainly accumulated in the spleen, kidney and lung, and the amount of gold in organs decreased with time (Figure 6B). Urine and feces were collected for cleared TMA‐GNC quantification. Within 24 h, ≈20% of injected gold was cleared out of mice, demonstrating the clearable capabilities of TMA‐GNC in vivo (Figure 6C). Next, routine blood examinations, serum biochemistry assays, and hematoxylin and eosin (H&E) staining of the major organs were performed to evaluate possible toxicities after the i.p. injection of TMA‐GNC. All hematological parameters of mice treated with TMA‐GNC were similar to those of the untreated mice (Figure 6D). No noticeable damage or inflammation was observed in the histological sections of organs (heart, lung, liver, spleen, and kidney) after TMA‐GNC administration (Figure 6E). We injected TMA‐GNC intraperitoneally at higher concentrations into mice and recorded the survival rate after 48 h. All mice (10/10) survived when the injection dosage was below 32 mg kg^−1^, and a survival rate of 70% (7/10) was identified at the injection dosage of 64 mg kg^−1^ (Figure 6F). In summary, TMA‐GNC was demonstrated to be highly compatible with blood, clearable after in vivo administration, and biologically safe at certain doses, suggesting TMA‐GNC could be used to reduce coronavirus disease in vivo.

### 2.5 Therapeutic Activity of TMA‐GNC in vivo

**Figure 7:**
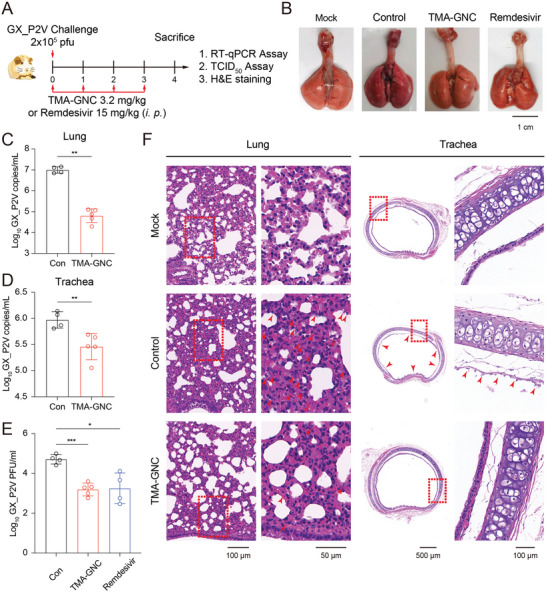
Anti‐coronaviral activity of TMA‐GNC in vivo. A) Schematic diagram of in vivo treatment of GX_P2V infection by nasal inhalation of TMA‐GNC in hamsters. The single dose of TMA‐GNC and remdesivir were i.p. administrated into the infected hamsters at 0, 1, 2, and 3 days post‐infection, respectively. The first treatments at 0 dpi were performed at 2 hours post the infection. B) Ex vivo images of lung tissues. Viral genome copies in the lung (C) and trachea (D) were determined by RT‐qPCR, respectively. E) TCID_50_ assay of virions harvested from lungs. F) Hematoxylin and eosin staining of lungs and tracheas.

We used the golden Syrian hamster model to evaluate the anti‐coronavirus activity of TMA‐GNC in vivo. Hamsters were challenged with GX_P2V (2 × 10^5^ pfu) by nasal inhalation. Hamsters were treated with TMA‐GNC (3.2 mg kg^−1^, corresponding to 25 mg/human) and remdesivir (15 mg kg^−1^, standard dosing) to determine their therapeutic efficacy. The animals were sacrificed 4 days post‐infection, and viral copies, infectious virus titres and pathologic changes were analyzed by RT‐qPCR assay, TCID_50_ assay and H&E staining (**Figure** **7**A). Post‐mortem analysis showed that the lung tissue of the TMA‐GNC‐treated hamster remained as smooth as that of the healthy lung, but the infected lung turned dark red in appearance, with palpable nodules scattered across the lung lobe (Figure 7B). Consistently, the suppression of GX_P2V viral genome copies in tissues, including the lungs and tracheas, was confirmed in TMA‐GNC‐treated hamsters. TMA‐GNC treatment diminished viral copies in lung tissues by ≈2–log_10_ value (Figure 7C) and the trachea by ≈0.5–log_10_ value (Figure 7D). We next examined viral virulence in lung tissues after treatment using the TCID_50_ assay. The results showed that both TMA‐GNC and remdesivir decreased the TCID_50_ values by ≈2–log_10_ value (Figure 7E and Figure S14, Supporting Information). However, the therapeutic dosage of remdesivir used in this study was 4.8 times higher than that of TMA‐GNC, reflecting the superior therapeutic activity of TMA‐GNC in vivo. To examine the severity of lung damage, we performed H&E staining of lung tissues and trachea. Thickening of the alveolar septum and a large amount of lymphocyte infiltration were observed in the control hamster lung. In contrast, the TMA‐GNC‐treated lungs exhibited improved morphology and milder infiltration (Figure 7F). In the trachea, massive loss or absence of mucosal epithelial cells was observed in the positive control group, whereas the TMA‐GNC‐treated group maintained a normal morphology. Altogether, we demonstrated that TMA‐GNC antagonized GX_P2V in vivo, as determined by the reduced viral titres, virulence and pathological damage after treatment.

## 3 Conclusion

In conclusion, TMA‐GNC was identified as an intrinsically broad‐spectrum anti‐coronaviral nanodrug with multiple mechanisms in inhibiting SARS‐CoV‐2 trVLP and SARS‐CoV‐2 pseudoviruses infection. TMA‐GNC inhibited the coronavirus by directly destroying the structural membrane, interfering with 3CL^pro^ enzymatic activity, and modulating antiviral immune responses. These mechanisms would help TMA‐GNC avoid loss of effectiveness owing to constantly emerging variants. We identified the efficacy of TMA‐GNC in inhibiting viral infection in vivo at a safe dose; however, this was the first step toward discovering metallic nanoparticles with the potential to deal with future endemic coronaviruses. Future efforts should balance the therapeutic outcomes and potential biosafety concerns in large animal models. In addition, our data showed that the engineering of surface ligands endowed gold nanomaterials with anti‐coronavirus capabilities. A similar approach could be used to engineer organic scaffolds, such as polymer nanoparticles or dendrimers, to develop antiviral macromolecules.

## Conflict of Interest

The authors declare no conflict of interest.

## Supporting information

Supporting Information

## Data Availability

The data that support the findings of this study are available from the corresponding author upon reasonable request.
