# Supplementary material for: Anti‐Coronaviral Nanocluster Restrain Infections of SARS‐CoV‐2 and Associated Mutants through Virucidal Inhibition and 3CL Protease Inactivation
Source: Adv Sci (Weinh). 2023 Feb 26;10(13):2207098. doi: 10.1002/advs.202207098 (PMC10161070; doi:10.1002/advs.202207098)
Supplement: Supplementary file 1 — Supporting Information [file ADVS-10-2207098-s001.pdf]

ADVANCED SCIENCE
Open Access

Supporting Information

for *Adv. Sci.*, DOI 10.1002/advs.202207098

Anti-Coronaviral Nanocluster Restrain Infections of SARS-CoV-2 and Associated Mutants through Virucidal Inhibition and 3CL Protease Inactivation

Hao Tang, Hongbo Qin, Shiting He, Qizhen Li, Huan Xu, Mengsi Sun, Jiaan Li, Shanshan Lu, Shengdong Luo, Panyong Mao, Pengjun Han, Lihua Song, Yigang Tong, Huahao Fan* and Xingyu Jiang**

Supporting Information

Anti-coronaviral Nanocluster Restrain Infections of SARS-CoV-2 and Associated Mutants through Virucidal Inhibition and 3CL Protease Inactivation

Hao Tang,† Hongbo Qin,† Shiting He, Qizhen Li, Huan Xu, Mengsi Sun, Jiaan Li, Shanshan Lu, Shengdong Luo， Panyong Mao, Pengjun Han, Lihua Song, Yigang Tong, Huahao Fan,* Xingyu Jiang**

† These authors contributed equally to this work.

* Corresponding author.

E-mail: tongyigang@mail.buct.edu.cn (Yigang Tong); fanhuahao@mail.buct.edu.cn (Huahao Fan); jiang@sustech.edu.cn (Xingyu Jiang)

Materials

Chemicals and agents

Gold chloride trihydrate ($HAuCl_4 \cdot 4H_2O$, cat no. 141105) was purchased from Aladdin. *N, N, N*-trimethyl(11-mercaptoundecyl) ammonium chloride (TMA, cat no. FT006) was purchased from Prochimia Surfaces. Reduced L-glutathione (GSH, cat no. G4251) was purchased from Sigma-Aldrich. SARS-CoV-2 Nucleocapsid antibody (3F9) (cat no. A02049-100) was purchased from GenScript. GAPDH monoclonal antibody (cat no. 60004-1-Ig) and HRP-conjugated AffiniPure Goat Anti-Mouse IgG (H+L) (cat no. SA00001-1) were purchased from Proteintech. SARS-CoV-2 $3CL^{pro}$ (cat no. CR76) were purchased from Novoprotein. The fluorogenic peptide Dabcyl-KNSTLQSGLRKE-Edans was synthesized by GeneScript. Britelite plus reporter gene assay system (cat no. 6066769) was purchased from PerkinElmer.

Cells, viruses, and animals

The African green monkey kidney (Vero E6) cells, human lung adenocarcinoma (Calu 3) cells, and buffalo green monkey kidney (BGM) cells were obtained from American Type Culture Collection (ATCC, Manassas, VA, USA). Cells were maintained in Dulbecco's Modified Eagle Medium (DMEM, HyClone, cat no. SH30243.01) that supplemented with 10% fetal bovine serum (FBS, PAN-Biotech, cat no. ST30-2602) and 1% antibiotic-antimycotic (Gibco, cat no. 15070063). The SARS-CoV-2-related pangolin coronaviruses GX_P2V (accession No. MT072864.1) was isolated from smuggled dead Manis javanica in 2017. Coronavirus GX_P2V was maintained and multiplied in Vero E6 cells. The GenBank accession number of PEDV and SADS-CoV was AF353511.1 and MT747188.1, respectively. The GenBank accession number of rotavirus was AF317123. Male or Female golden hamsters (8-9 weeks) were purchased from Charles River Laboratories (Beijing, China). The GX_P2V associated animal experiments were carried out in BSL-2 laboratory of the Fifth Medical Center (Chinese PLA People's Liberation Army General Hospital) with the approval of experimental animal welfare and ethics (Approval ID: IACUC-2018-0020). Female BLAB/c mice (4-5 weeks) were purchased from Huafukang Biotechnology (Beijing, China). *In vivo* experiments that aimed to evaluate the biodistribution and biosafety properties of TMA-GNC were conducted with the permission of the Institutional Animal Care and Use Committee of the Southern University of Science and Technology (SUSTC-2019-132).

Synthesis and characterisation of anti-coronavirus gold nanoclusters

TMA-GNC was synthesised through a hydrothermal reduction approach. Briefly, the aqueous solution of TMA (25 mM, 2 mL) and GSH (100 mM, 0.5 mL) were mixed into a round-bottom flask that contained 6.5 mL Milli-Q water. The flask was then placed into an oil bath (70 °C) under vigorous stirring (600 rpm). Then, a 1 mL aqueous solution of auric acid (25 mM, 1mL) was added to the mixture to start the reaction, and the speed of stirring was increased to 1000 rpm. After reaction for 24 h, the GNC was purified by dialysing (molecular weight cut-off: 3500 Da) against Milli-Q water to remove excess TMA and GSH. The obtained GNC was condensed by ultrafiltration (Pall corporation, cat no. FG8207) and stored at 4 °C for further study.

The hydrodynamic diameter and zeta potential of TMA-GNC were measured by the Nano-ZS Zetasizer (Malvern Instruments). The high angle annular dark-field STEM (HAADF-STEM) images and elemental analysis of TMA-GNC were conducted with the Themis G2 (FEI) at the accelerating voltage of 300 kV. The ratio of TMA and GSH on the surface of GNC was determined by ^{1}H NMR analysis after etching the gold core with iodine. X-ray photoelectron spectroscopy (XPS) analysis was conducted on the ESCALAB 250Xi instrument (Thermo Scientific) with an excitation source of Al Kα X-ray radiation (1486.6 eV). The concentration of gold element was determined by the inductively coupled plasma mass spectrometry ICP-MS (7700X, Agilent).

Viral infection assay *in vitro* and determination of EC_{50}

To test the anti-GX_P2V ability of TMA-GNC, Vero E6 cells were inoculated into 96-well plate (4×10^4 cells/well) and incubated overnight. GX_P2V at the MOI of 0.01 was mixed with TMA-GNC in gradient dilutions (final concentrations ranged from 0.0078 μM to 2 μM) and were then added to the cells in duplicate. After 2 h incubation, the inoculum was removed and replaced by fresh media with the same concentration of TMA-GNC contained. At 48 h post-infection in the presence of TMA-GNC, cytopathic effects were observed by microscope. Cells were collected for the RT-qPCR analysis, and the EC_{50} was determined according to the RT-qPCR results. Antiviral assays of Nirmatrelvir (PF-07321332), Remdesivir (GS-5734), and Aluvia were performed as control. In the SARS-CoV-2 trVLP assay, SARS-CoV-2 trVLP (MOI of 0.01) and TMA-GNC (final concentrations ranged from 0.0078 μM to 2 μM) were mixed and directly added into the Caco-2-N cells without incubation. The following procedures remained the same to that of GX-P2V assays.

Viral RNA extraction, reverse transcription, and real-time quantitative PCR (RT-qPCR)

After harvesting the cells and the supernatant, the total viral RNA was extracted and purified using Flying Shark® Tissue& Cell RNA Kit (cat no. RNE11, Nobelab Biotech, Beijing, China) and AxyPrep™ Body Fluid Viral DNA RNA Kit (cat no. 05921KC5, Corning Life Science, Wujiang, China) in accordance with manufacturer's instructions. Reverse transcription was performed using Hifair II 1st Strand cDNA Synthesis Kit (Yeasen Biotech, Shanghai, China) to obtain viral cDNA. To perform the real-time quantitative PCR (RT-qPCR) assay, 1 μL viral cDNA was mixed with 10 μL Hieff qPCR SYBR Green Master Mix, 0.4 μL forward primer, 0.4 μL reverse primer and 8.2 μL water. The RT-qPCR was performed in QuantStudio 1 Real-Time PCR detection system (Applied Biosystems, CA, USA) with two-stage SYBR Green method. The procedure was as follows: 95 °C for 10 minutes, then 95 °C for 10 seconds and 60 °C for 30 seconds with 40 cycles; melting curve stage: 95 °C for 15 seconds, then 60 °C for 1 minute and 95 °C for 1 second. The primer sequences were listed as followed:

GAPDH-F, 5'-AGCCTCAAGATCATCAGCAATG-3'.

GAPDH-R, 5'-ATGGACTGTGGTCATGAGTCCTT-3'.

GX_P2V-F, 5'-GGTGATTGCCTTGGTGATATTG-3'.

GX_P2V-R, 5'-GCAAGTAGTGCAGAAGTGTATTG-3'.

To measure the viral gene copies number, the PCR product was inserted into a vector to synthesize standard plasmid. After determining the copy number, the plasmid was serially diluted (10^{-3} ~ 10^{-9}) for RT-qPCR analysis. The standard curve was generated according to the copy numbers and cycle threshold values.

Determination of half-cytotoxic concentration (CC_{50})

Vero E6 cells were inoculated into 96-well plate (4×10^4 cells/well) and incubated overnight. TMA-GNC with different dilutions (0.0078-2 μM) were introduced to the cells to incubate for 48 h. After incubation, each well was treated with 20 μL of resazurin (Promega) for 2 h and the absorbance at 570 nm was measured using microplate reader. The cytotoxity was calculated by: Inhibition (%) = $1 - (OD_{TMA\text{-}GNC}/OD_{control}) \times 100$ %. The calculation of CC_{50} was based on the results of inhibition.

Pseudovirus Inhibition Assay

SARS-CoV-2 pseudoviruses were generated using the Vesicular Stomatitis Virus (VSV) pseudovirus packaging system[50]. Plasmids expressing various S protein variants of SARS-CoV-2 were synthesized by RuiBiotech (Beijing, China). Briefly, TMA-GNC was mixed with pseudoviruses of SARS-CoV-2 wild type or mutant strains for a co-incubation in the cell incubator. The concentration of TMA-GNC was 3 μM and 6 μM. After 90 min incubation, resuspended BHK21-ACE2 cells (5×10^4 cells/well) were added into the each well of the TMA-GNC/pseudoviruses mixtures. Cells were collected at 24 h post-incubation, washed with PBS, and fully lysed. The activity of firefly luciferase was measured using the firefly luciferase reporter gene assay kit (Yeasen, cat no. 11401ES76) for the determination of inhibition rate of TMA-GNC against the pseudoviruses. The relative light unit (RLU) was recorded by a microplate reader (Bio-Rad, USA).

Plaque assay

To perform the plaque assay, Vero E6 cells were pre-seeded in a 6-well plate. The supernatant of infected cells with 10-fold dilution (10^{-1} ~ 10^{-6}) was added to the plate (1 mL per well) and incubated for 1 h. After the free viruses were discarded, the cells were overlaid with medium containing 1% agarose to prevent cross infection. At 3 days, cells were fixed with 4% paraformaldehyde for 2 h and stained with crystal violet for 10 minutes. The plaques were count after the crystal violet was washed with water. The titer was calculated as follow: Titer (PFU/mL) = the number of plaques × dilution multiple.

Western blotting

Western blotting was performed to detect the production of GX_P2V nucleocapsid protein. After protein quantification, 20 μL of the samples with the same protein concentration were loaded on a 12% SDS-PAGE gel for electrophoresis (80 V for 30 min and 120 V for 60 min). After electrophoresis, proteins were transferred to a polyvinylidene (PVDF) fluoride membrane (15 V for 60 min). The PVDF membrane was blocked by 5% skim milk (TBST) for 1 h at room temperature. After washing out of the blocking reagent, the antibody against nucleocapsid protein of anti-SARS-CoV-2 N protein (Genscript, USA) and GAPDH of anti-GAPDH (Proteintech, USA) were used at 1:200 and 1:2000 dilutions, respectively. After 2 h incubation at room temperature, the antibody was removed thoroughly, and the second antibody of HRP-conjugated AffiniPure Goat anti-mouse IgG (H+L) diluted at 1:10000 was used for another 2 h incubation. Then SuperSignal® West Femto Maximum Sensitivity Chemiluminescent Substrate (Thermo Scientific, USA) was used for imaging.

Time-of-addition assay

Time-of-addition assay was performed to determine at which stage of life cycle TMA-GNC exerted its inhibition effect. Vero E6 cells were seeded into 48-well plate (1×10^5 cells/well) and incubated overnight. GX_P2V at the MOI of 0.01 and TMA-GNC at concentrations of 1.5 μM and 3.0 μM were mixed respectively. The mixtures were added to the cells for 2 h incubation to allow viral attachment and internalization. Then the viruses were removed, and the cells were washed with PBS. The cells were re-supplemented with fresh media and cultured for another 48 h. For post-entry experiment, the cells were first infected by GX_P2V at the MOI of 0.01 for 2 h. After the infection, viruses were removed, and the cells were wash with PBS. The cells were then cultured with the media containing TMA-GNC at 1.5 μM and 3.0 μM for 48 h. The cells were collected for RT-qPCR analysis to measure the intracellular viral production.

Viral attachment assay

Vero E6, Calu 3 and BGM cells were seeded into a 24-well plate (5×10^5 cells/well) and incubated overnight. 3 μM of TMA-GNC was incubated with GX_P2V (MOI=10) at 4 ^{o}C for 2 h to allow interaction, then cells were treated with the mixture for 2 h incubation at 4 ^{o}C. After GX_P2V had been attached to cells, the unbind GX_P2V was removed, and cells were washed with PBS for 3 times and collected for RT-qPCR analysis.

Cyro-EM

Inactivated GX_P2V (1×10^6 PFU/mL) were incubated with or without TMA-GNC (1.5 μM) for different time intervals (30, 60, 120, and 180 min). The mixtures were adsorbed on carbon-coated grids using the Vitrobot (FEI). Images were taken under the liquid nitrogen with the Titan Krios G3i (D3845) at a voltage of 300 kV.

***In vitro* FRET assay of SARS-CoV-2 3CL^{pro} inhibition**

The TMA-GNC was first diluted to successive concentrations, and each TMA-GNC solution was thoroughly mixed with SARS-CoV-2 3CL^{pro}. The mixtures were incubated at 4 °C for 2 h. The mixed samples were added to a 96-well plate and followed by the addition of fluorescent substrate solution into each well to start the reaction. The final concentration of 3CL^{pro} and fluorescent substrate reached 1 µM and 10 µM, respectively. The final concentration of TMA-GNC ranged from 0.0003 µM to 24 µM. Controls were set as (1) 3CL^{pro} plus fluorescent

substrate without TMA-GNC addition, and (2) fluorescent substrate plus TMA-GNC without $3CL^{pro}$ addition. The changes in fluorescence intensity (Ex: 340 nm, Em: 488 nm) within 1 h were detected by a microplate reader. The measured values were averaged (n = 3). The reaction rate was determined by the digestion of fluorescent substrate by $3CL^{pro}$ in the presence of different concentrations of TMA-GNC. The inhibition rate of $3CL^{pro}$ in the presence of diverse concentrations of TMA-GNC was calculated by comparing the corresponding reaction rate with the control (1). That was, Inhibition rate = 100% - relative activity of the enzyme at different TMA-GNC concentrations × 100%). The IC_{50} value was determined using non-linear fitting by GraphPad Prism 8 software.

Bio-layer interferometry (BLI) analysis

The BLI experiments were performed on the OctetRed384 (ForteBio) to determine the binding kinetics between SARS-CoV-2 $3CL^{pro}$ and TMA-GNC. Briefly, the alginate coated biosensors (AR2G, cat no. 18-5092) were first dipped into the aqueous solution of TMA-GNC (1.2 μM) to attach GNC onto the sensors through electrostatic interaction. The loaded TMA-GNC did not detach from the biosensors in buffers we used in this study except in the glycine solution (pH = 2.0, Cytiva, cat no. BR100355). The biosensors were then dipped into PBS solutions of SARS-CoV-2 $3CL^{pro}$ (62.5, 125, 250, 500, and 1000 nM) for association (100 sec) and dissociation (150 sec). The data were corrected with blank curves and fitted with Octet evaluation software using the 1:1 binding mode. To determine the binding affinities under different pH conditions, SARS-CoV-2 $3CL^{pro}$ was diluted with buffers with a pH value ranging from 7.4 to 4.5. To determine the binding sites of TMA-GNC towards SARS-CoV-2 $3CL^{pro}$, the $3CL^{pro}$ (1000 nM) got trypsinisation overnight at 37 °C before the BLI experiments. Enriched peptides were detached from the surface of TMA-GNC by using iodine to dissolve gold and further desalted through StageTip C18 (Thermo) before MS analysis. The sequence identification by MS was performed with the Orbitrap Fusion (Thermo Fisher Scientific). Proteome Discovery (Version 2.4) software (Thermo Fisher Scientific) was applied to search the raw data against SARS-CoV-2 $3CL^{pro}$ fasta files (downloaded on Dec 13, 2021). 1% of the false discovery rate was set for peptide identification.

Safety evaluation

Hemolysis assay

Erythrocytes were obtained by centrifuging fresh rat blood samples (1500 rpm, 15 min) and washed with saline three times. Suspended erythrocytes (4%, v/v) were incubated with TMA-

GNC (0.75, 1.5, 3, 6, 12, 24, 36, 48, 60 μM) for 4 h at 37 °C, while water was used as the positive control. The mixture solutions were centrifuged at 12,000 rpm for 15 min, and the absorbance at 540 nm of supernatants was measured by the microplate reader (Spark, Tecan).

Biodistribution and toxicity evaluation *in vivo*

To investigate the biodistribution and clearance of TMA-GNC post-injection, the GNC was administrated into BLAB/c mice at 10 mg/kg intraperitoneally. At designed time points (24, 48, and 72 h), mice were sacrificed, and major organs (heart, liver, spleen, lung, and kidney) were collected. Urine and faeces of the mice were collected using the metabolism cages. All biological samples were lysed with freshly prepared aqua regia overnight and followed by heating for efficient nitrolysis and complete removal of aqua regia. Samples were re-dissolved with 2% (v/v) HNO_3 and applied to the ICP-MS measurements for the qualitative determination of gold. Blood was sampled from the eyes of mice. The whole blood (~50 μL) was applied to routine tests, and the rest of the blood samples were centrifuged at 3000 rpm and 4 °C for 10 min to obtain the serum. Biochemical indicators in all serum samples were measured with an automatic biochemistry analyser MS480 (MedicalSystem, China). Tissues of major organs were also subjected to H&E staining for pathological analysis. To determine the potential toxicity of TMA-GNC at a high dosage, mice were treated with TMA-GNC with a concentration of 3.2, 16, 32, and 64 mg/kg (n = 10), and the survival rate was recorded.

Antiviral evaluation in the golden hamster model

Fifteen golden hamsters (7 females and 8 males, 8–9-week-old) were divided into four groups. Golden hamsters in the groups above were challenged by 2×10^5 pfu GX_P2V on 0 dpi with a nasal infection. The TMA-GNC treatment group consisted of two females and three males, which were intraperitoneally administered with TMA-GNC diluted in distilled water at a dose of 3.15 mg/kg on 0, 1, 2, 3 dpi. Remdesivir group, comprising two males and two females, was designed as a positive drug control. In this group, remdesivir was injected intraperitoneally at a dose of 25 mg/kg on 0, 1, 2, and 3 dpi. The dosage of remdesivir was referred to in previous research[12]. The negative control group was performed by intraperitoneal injection with the same amount of PBS per day. Animals in each group were sacrificed and anatomised at 4 dpi. The lung tissues were obtained by anatomy and photographed. Partial lung tissues were collected to quantify the viral yield by $TCID_{50}$ assay and RT-qPCR analysis. Lung tissues soaked in PBS were ground to harvest homogenate using a freeze grinder (JingN·9548R; Hoder, Beijing, China), then centrifuged at 12000 ×g for 3

min at 4 °C. For the $TCID_{50}$ assay, the supernatant was serially diluted (10^{-1} - 10^{-3}), and dilutions were inoculated to a 96-well plate pre-seeded with Vero E6 cells in duplicate. After 72 h incubation, the CPE of each well was observed to calculate $TCID_{50}$. For RT-qPCR analysis, viral RNA was extracted from supernatant using Flying Shark® Tissue& Cell RNA Kit (Cat No. RNE11, Nobelab Biotech, Beijing, China). The following treatments were as described above. Tissues of the lung and trachea were subjected to H&E staining for pathology analysis.

RNA-seq analysis

Four experimental groups were set up: (i) Caco-2-N cells were cultured for 48 h without virus infection or treatment with TMA-GNC; (ii) Caco-2-N cells were treated with 3 μM of TMA-GNC and cultured for 48 h; (iii) Caco-2-N cells were infected with SARS-CoV-2 trVLP at MOI = 0.01 for 2 h and then continued to be cultured for 48 h; (iv) Caco-2-N cells were treated with TMA-GNC at the same concentration as (ii), and infected with SARS-CoV-2 trVLP at MOI = 0.01 for 2 h and then cultured for 48 h. Total RNA was extracted using Trizol, amplified, and RNA libraries were built, purified, and sequenced (Annoroad Gene Technology, Beijing, China). Sequences were corrected and then mapped using TopHat2 and bowtie2. Each gene was counted using HTSeq-count. Pairwise comparisons were performed, and the differentially expressed genes were analysed using an R packaged cluster profiler.

Statistical analysis

Statistical analyses were conducted using GraphPad Prism 8. Unpaired t tests were used to evaluate the changes in viral titres and RNA content after drug treatments. P values ≤ 0.05 was regarded as significant for the analysis and marked with asterisks: * $P \leq 0.05$, ** $P \leq 0.01$, *** $P \leq 0.001$. For the determination of EC_{50}, CC_{50} and IC_{50} value, nonlinear fit analysis was performed.

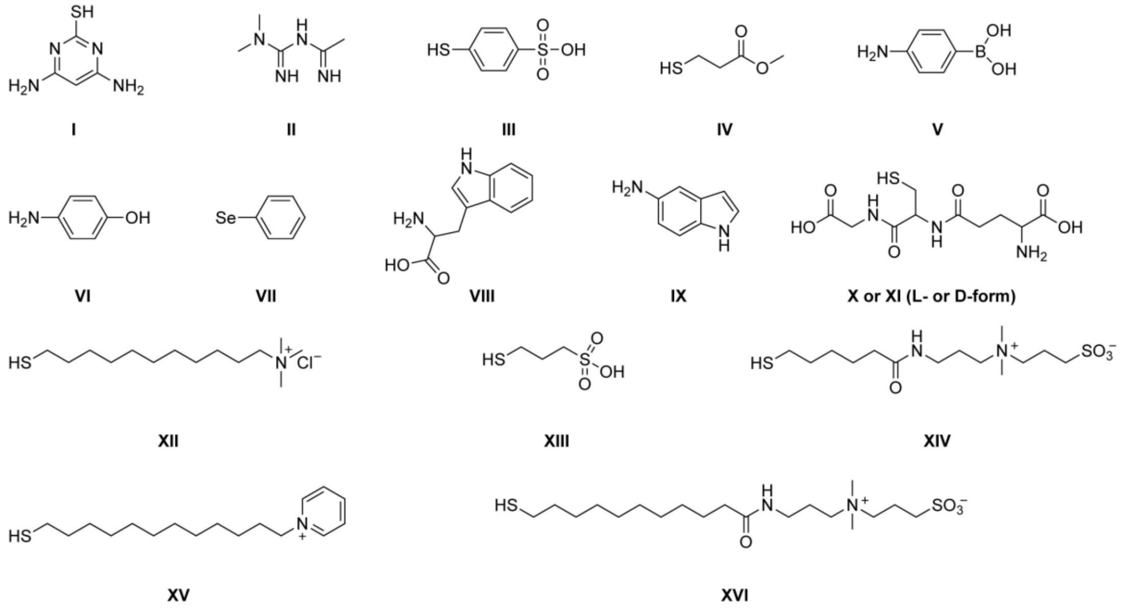

Figure S1. Chemical structures of surface ligands used to modify the gold nanomaterials to screen potential anti-coronaviral nanomedicine. Ligands for each gold nanomedicines (1-19) were listed. 1: I, 2: II, 3: I+II, 4: III, 5: IV, 6: V, 7: VI, 8: VII, 9: VIII, 10: IX, 11: X+I, 12: XI+I, 13: X+XII, 14: X+I+VII, 15: VIII, 16: XIV, 17: XV, 18: XIV+XV, 19: XVI.

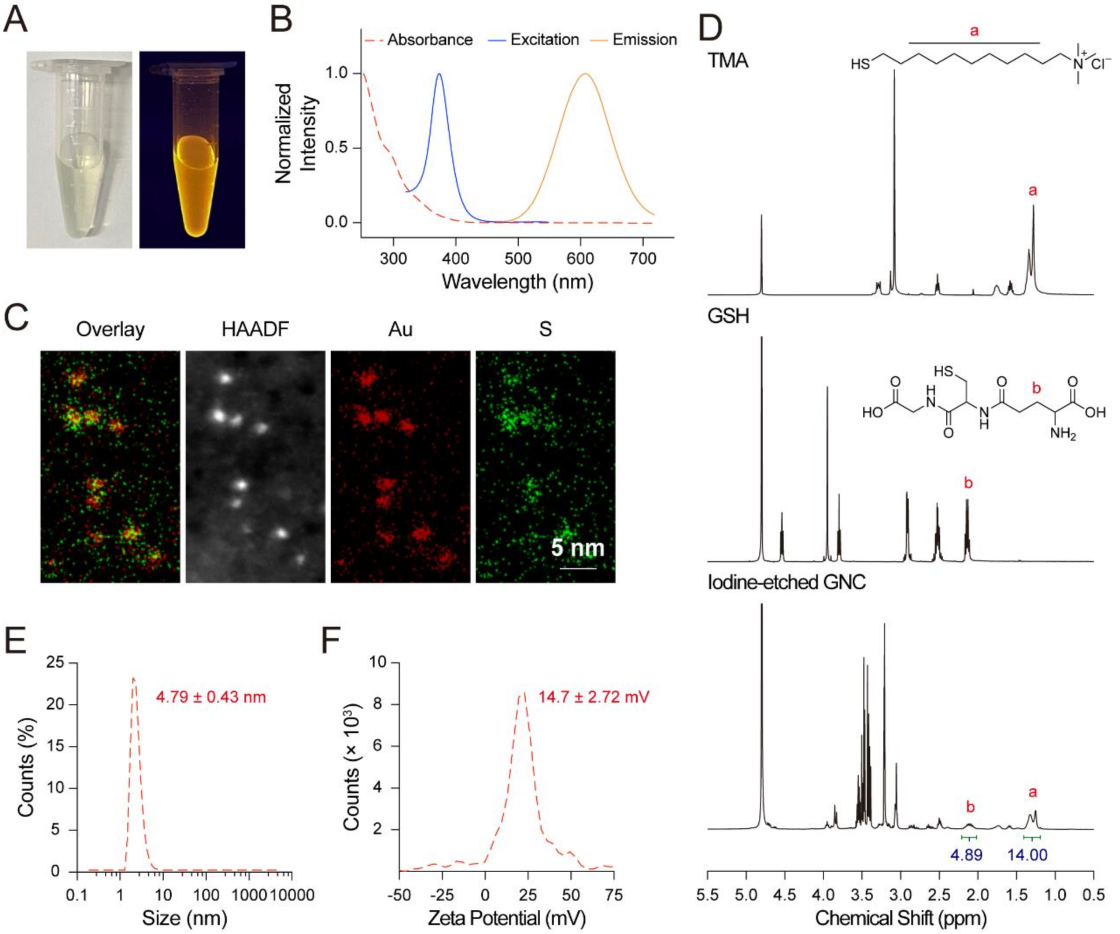

Figure S2. (A) The appearance of TMA-GNC under the daylight (left) and UV light (365 nm, right). (B) UV-Vis spectrum and fluorescence spectrum of excitation and emission of TMA-GNC. (C) The energy-dispersive X-ray spectrometry (EDS) elemental analysis of TMA-GNC. (D) ^{1}H NMR spectrum of TMA-GNC. The free ligands (TMA and GSH) were dissolved in D_2O, and the iodine-etched GNC was dissolved in MeOD. (E) Hydrodynamic diameter and (F) zeta potential of TMA-GNC in aqueous solution.

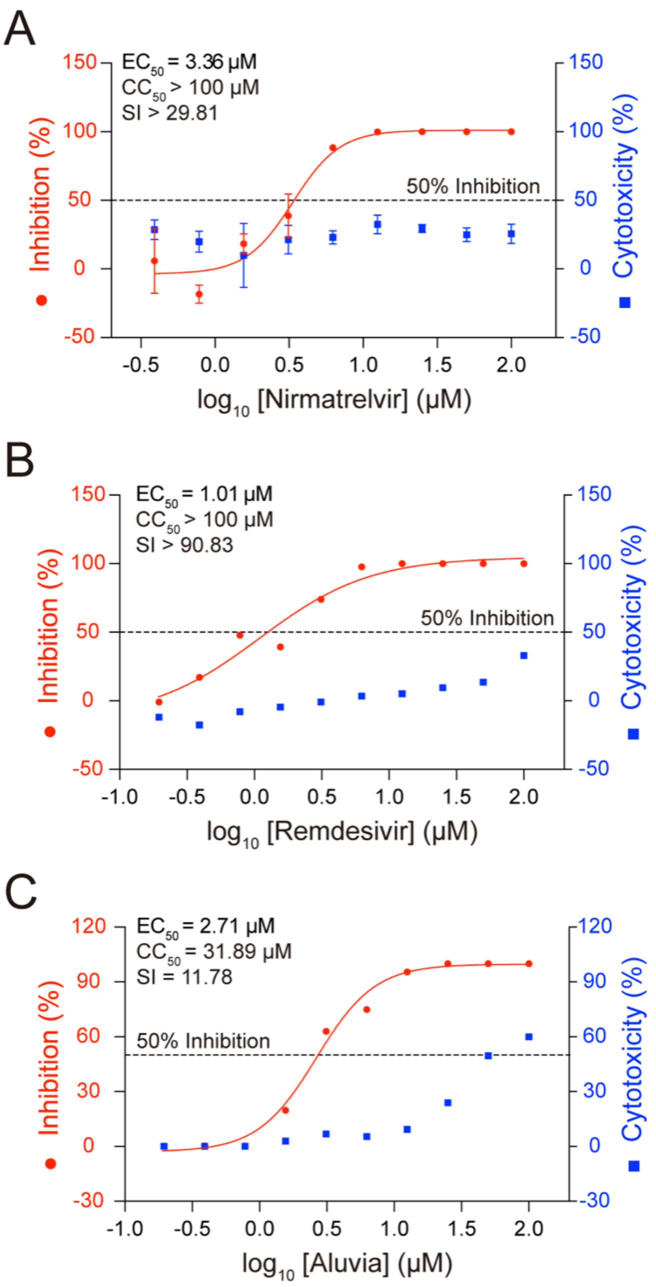

Figure S3. Evaluation of anti-GX_P2V efficacy of (A) Nirmatrelvir (PF-07321332), (B) Remdesivir (GS-5734), and (C) Aluvia (Lopinavir and Ritonavir).

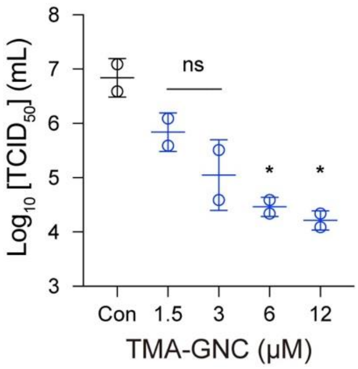

Figure S4. Median tissue culture infectious dose ($TCID_{50}$) value of produced virions of GX_P2V after treatment with TMA-GNC.

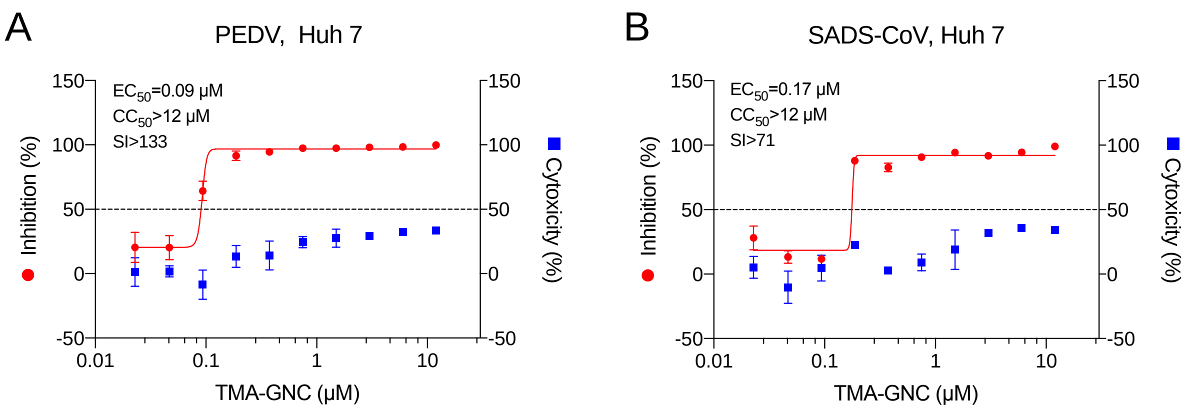

Figure S5. Inhibition analysis of PEDV and SADS-CoV infection on Huh-7 cells by different doses of TMA-GNC.

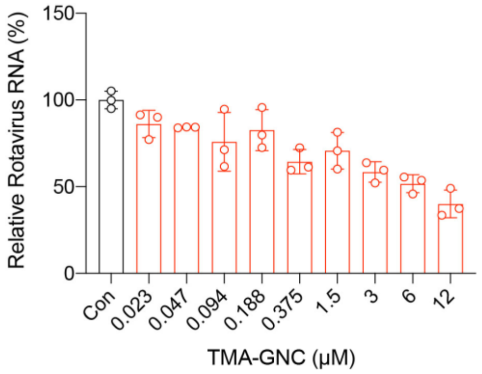

Figure S6. Inhibition analysis of rotavirus infection on Huh-7 cells by different doses of TMA-GNC.

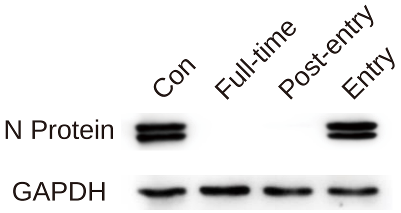

Figure S7. WB analysis inhibition of GX_P2V infection by TMA-GNC during full-time, entry, and post-entry processes.

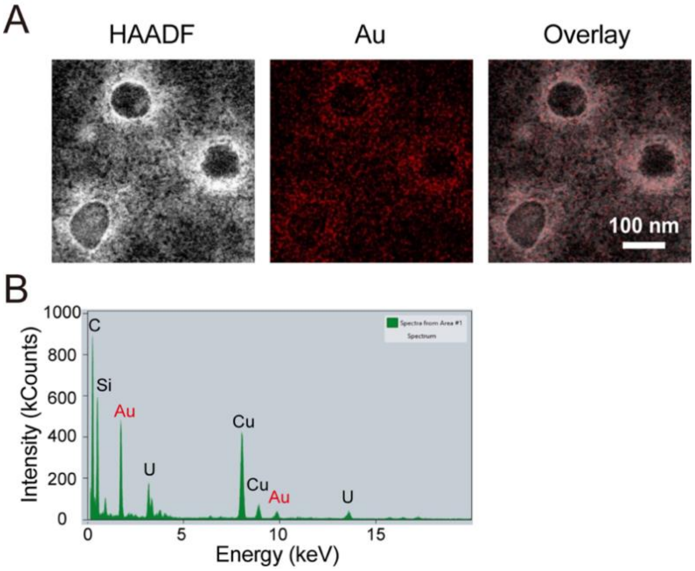

Figure S8. EDS elemental analysis of GX_P2V after co-incubation with TMA-GNC.

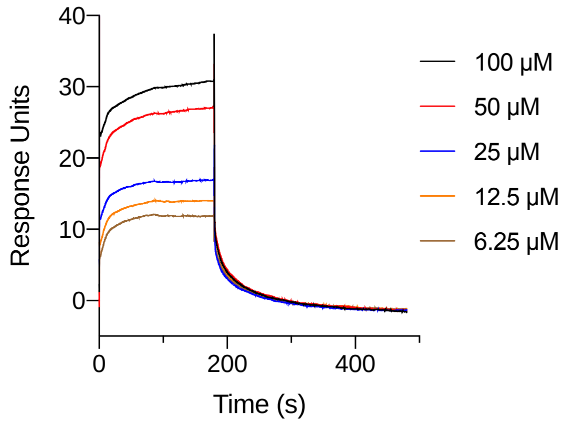

Figure S9. The binding kinetics of SARS-CoV-2 3CL^{pro} and ebselen. The K_D was determined to be 6.8×10^{-6} M.

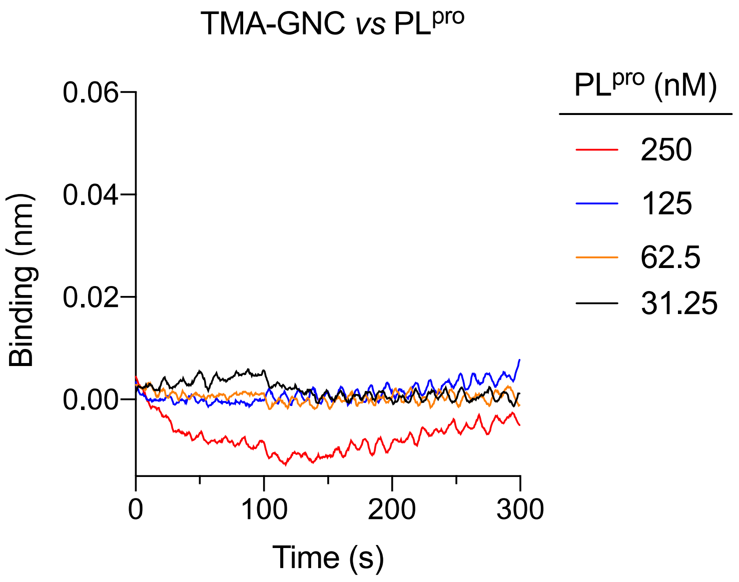

Figure S10. The binding kinetics of TMA-GNC and SARS-CoV-2 PL^{pro}.

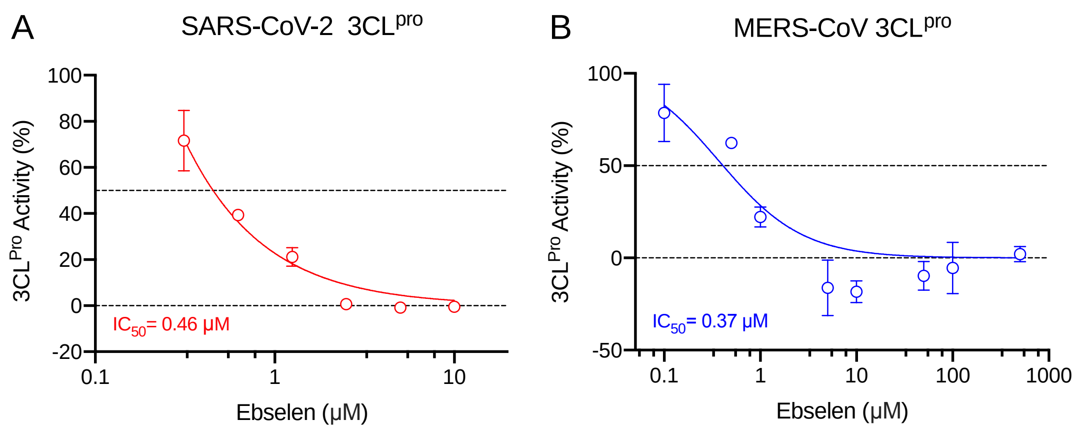

Figure S11. Dose-response curves for the inhibition of (A) SARS-CoV-2 3CL^{pro} and (B) MERS-CoV 3CL^{pro} by ebselen.

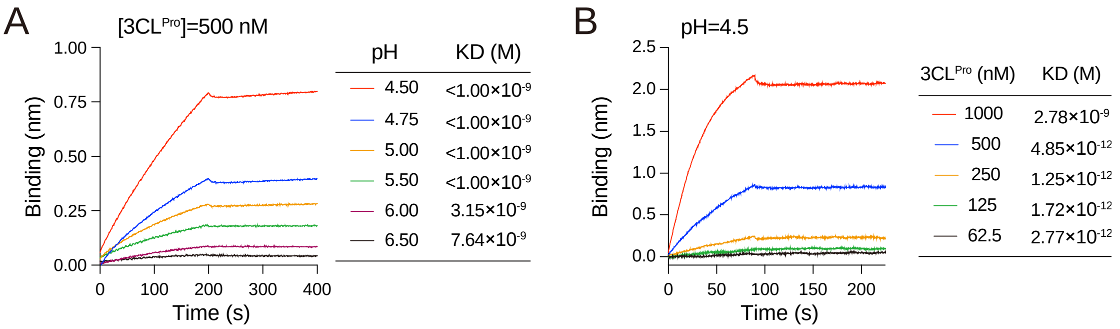

Figure S12. (A) The binding kinetics of TMA-GNC and SARS-CoV-2 3CL^{pro} in buffers at different pH. The concentration of 3CL^{pro} was 500 nM. (B) The binding kinetics of TMA-GNC and SARS-CoV-2 3CL^{pro} in an acidic buffer (pH = 4.5).

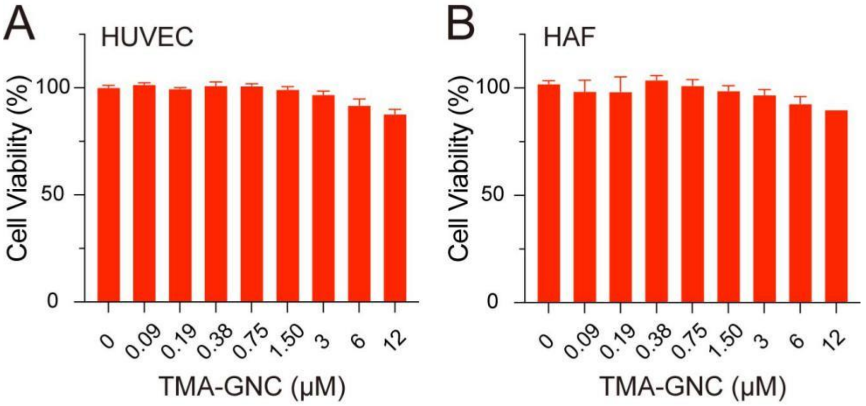

Figure S13. Cell cytotoxicity assays normal cell lines, including (A) human umbilical vein endothelial cells (HUVEC) and (B) human aortic fibroblasts (HAF) after treatment with TMA-GNC for 24 h.

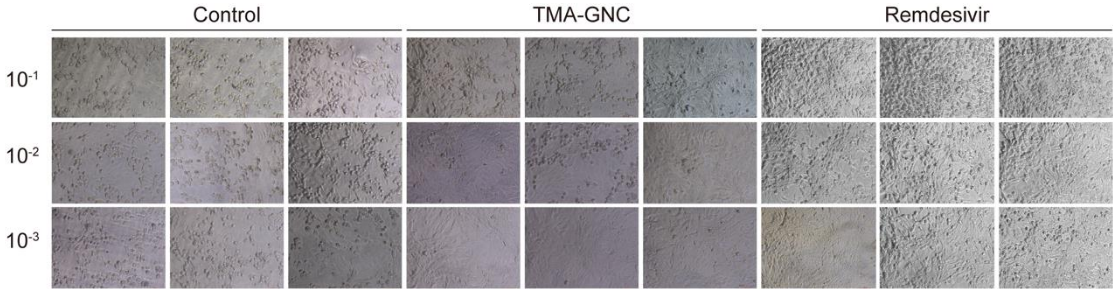

Figure S14. $TCID_{50}$ assay of the lungs of the control, TMA-GNC, and Remdesivir groups.
